# ConKit: a python interface to contact predictions

**DOI:** 10.1093/bioinformatics/btx148

**Published:** 2017-03-22

**Authors:** Felix Simkovic, Jens M H Thomas, Daniel J Rigden

**Affiliations:** Department of Biochemistry, Institute of Integrative Biology, University of Liverpool, Liverpool, UK

## Abstract

**Summary:**

Recent advances in protein residue contact prediction algorithms have led to the emergence of many new methods and a variety of file formats. We present *ConKit*, an open source, modular and extensible Python interface which allows facile conversion between formats and provides an interface to analyses of sequence alignments and sets of contact predictions.

**Availability and Implementation:**

*ConKit* is available via the Python Package Index. The documentation can be found at http://www.conkit.org. *ConKit* is licensed under the BSD 3-Clause.

**Supplementary information:**

Supplementary data are available at *Bioinformatics* online.

## 1 Introduction

Residue–residue contact predictions are becoming an increasingly popular and dynamic field of bioinformatics research as well as source of information in structural biology. Over recent years, great advances have been made to facilitate highly accurate predictions (e.g. Jones *et al.*, 2015; Marks *et al.*, 2011), which enabled these predicted contacts to, for example, guide accurate structure predictions (e.g. Michel *et al.*, 2014; Ovchinnikov *et al.*, 2015), identify functional sites (e.g. Grigolon *et al.*, 2016; Hopf *et al.*, 2012; Parente *et al.*, 2015), or predict modes of protein interaction (e.g. Hopf *et al.*, 2014; Ovchinnikov *et al.*, 2014).

The many prediction algorithms and pipelines currently available have adopted a variety of different file format conventions. Additionally, metapredictors rely on file conversions to either combine various predictions, or standardize the output in their chosen style. These various file formats lead to a dilemma for software developers writing tools to utilize predicted contacts. Although a standardized format—Casp RR—exists, it has not been widely adopted. Therefore, software developers must either insist on a given format for their tools, or develop an extensive library of conversion algorithms to handle multiple formats.

Users of contact prediction methods are often faced with the challenge of estimating the quality of a prediction. In co-evolution based methods, the quality typically depends on the Multiple Sequence Alignment depth (Jones *et al.*, 2015; Ovchinnikov *et al.*, 2015). However, the quantification of this depth, commonly referred to as number of effective sequences, and other important measures, such as the sequence coverage in the alignment, often requires further software packages or manual method development.

With this motivation, *ConKit* was developed to satisfy many of the issues outlined earlier and to provide additional functionality useful to a variety of software developers and users.

## 2 Materials and methods

*ConKit* is a cross-platform package written in the Python programming language. It is based in part on the *NumPy* (Oliphant, 2015), *SciPy* (http://www.scipy.org), *BioPython* (Cock *et al.*, 2009) and *matplotlib* (Hunter, 2007) packages. *ConKit*’s modular design enables it to have numerous applications as a standalone package. It is currently made up of four distinct packages—the data model, input/output structure, plotting facility and command-line application wrappers. *ConKit* also easily integrates into larger software packages and it is already distributed with *CCP4* v7.0.032 (Winn *et al.*, 2011) and *CCP-EM* beta (update January 7, 2017) (Wood *et al.*, 2015).

### 2.1 Data model

The underlying data model in *ConKit* stores contact information in a three-tier hierarchy, which provides easy access to the contact information stored within. At its lowest level, *ConKit* stores individual contact pairs in the Contact class. All Contact instances are combined and held in a ContactMap class, which provides routine functions to handle all contacts in a single prediction. At the top level, *ConKit* allows users to store multiple ContactMap instances in the ContactFile class. Each tier has attributes and functions relating to the data stored within, such as the sequence attribute in the ContactMap class, which allows users to easily associate a sequence with a contact prediction.

Alongside the data model for contact information, a SequenceFile hierarchy was implemented. Although *BioPython*’s SeqIO and AlignIO packages (Cock *et al.*, 2009) already provide such a data structure, *ConKit's* hierarchy enables customized interactions for the models. Two tiers are currently implemented, with the SequenceFile class storing one or more Sequence classes.

Both hierarchies provide storage, modification and analysis methods. For example, a ContactMap instance allows users to calculate the precision value of a given contact map when compared against the contacts extracted from a protein structure (Morcos *et al.*, 2011). This feature is essential for assessing the quality of contact predictions when structural information is available (Jones *et al.*, 2015; Monastyrskyy *et al.*, 2016). It also has potential value in scoring the quality of *ab initio* models based on the number of long-range contacts fulfilled in a model (de Oliveira *et al.*, 2016; Ovchinnikov *et al.*, 2017).

In comparison, a SequenceFile instance enables users to calculate the alignment depth, a key estimate for assessing the usefulness of an alignment in co-evolution based predictions (Monastyrskyy *et al.*, 2016). A SequenceFile instance also provides the functionality to determine the sequence coverage in the stored alignment, which can prove useful when trimming alignments to the core region of a protein domain.

### 2.2 Input/output

Manually constructing a data model in *ConKit* is generally not necessary. Four sequences and 17 contacts file format parsers have been implemented to allow read and write functionality. Importantly, the modular design of *ConKit* allows for an easy addition of new file format parsers in the future. In general, the two methods, read() and write(), are the access points to the parsers. To make file format conversions more accessible a third notable I/O function has been implemented, namely convert(), which acts as a wrapper encapsulating read() and write(). For a full list of available file formats, refer to *ConKit*’s documentation.

### 2.3 Data visualization

**Fig. 1:**
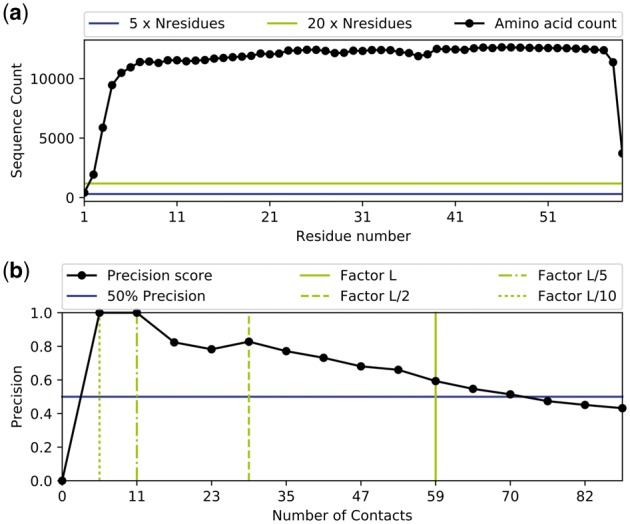
Data visualization in *ConKit*. **(a)** Sequence coverage in an example Multiple Sequence Alignment. **(b)** Precision score evaluation of a contact prediction file at various contact cutoff values where *L* = sequence length (rounded down to the nearest whole number of contacts). Data for both examples are based on PDB entry: 1DTX

Besides the analysis functionality outlined previously, ConKit also provides an interface for data visualization. It uses the *matplotlib* (Hunter, 2007) package and *ConKit*’s data models to extract and visualize data. Using the *matplotlib* package enables native support for many file formats, such as Portable Network Graphics or Scalable Vector Graphics. In *ConKit*, all plots are created via Python classes, thus enabling full customizability via simple class attribute setting. *ConKit* provides plotting classes for both SequenceFile and ContactMap hierarchies, such as the SequenceCoverageFigure class to illustrate the sequence coverage (Fig. 1a) in a Multiple Sequence Alignment or the ContactMapFigure for the commonly used contact map visualization (Supplementary Fig. S1). *ConKit* also provides the PrecisionEvaluationFigure class for a stepwise evaluation of precision scores when comparing a predicted contact map to protein structure (Fig. 1b). For a full list of available plots alongside usage examples, refer to the documentation.

### 2.4 Command-line application interface

Considering the number of different features of *ConKit*, we believe that the command-line application interface could be particularly useful to create metapredictor pipelines. To date, these wrappers comprise the following executables: *HHblits* (Remmert *et al.*, 2012); *Jackhmmer* (Johnson *et al.*, 2010); *HHfilter* (Remmert *et al.*, 2012); *CCMpred* (Seemayer *et al.*, 2014); *PSICOV* (Jones *et al.*, 2012); and *bbcontacts* (Andreani and Söding, 2015). All wrappers are based on the AbstractCommandline class in *BioPython*, and thus a fully working version of the package is required for this *ConKit* sub-package.

## 3 Usage

*ConKit* can be used in two distinct ways. To access all features, users do require some familiarity with the Python programming language. All packages outlined earlier are available via Python’s import system and relevant classes are exposed. To circumvent this requirement for non-programmers and make *ConKit* a more general tool, pre-defined routines in the form of scripts are automatically installed giving the general user access to *ConKit*’s key features from the command line. All scripts are written in Python making them operating system independent. All scripts have the prefix conkit and a one-word suffix based on its function. For example, conkit-msatool and conkit-convert can be used to analyse alignments and convert contact prediction files, respectively, while conkit-precision calculates the precision value given a contact prediction, the corresponding sequence and a protein structure. The conkit-plot script exposes *ConKit*’s plotting package for simple figure generation. For a full list of available scripts, refer to *ConKit*’s documentation.

## 4 Conclusions

We present *ConKit*, an extensible and modular Python interface for handling and manipulating residue–residue contact predictions, multiple sequence alignments and contact maps. Its core functionality is enhanced by the provision of command line scripts and application wrappers.

## Supplementary Material

Supplementary Data
