# Supplementary material for: ConKit: a python interface to contact predictions
Source: Bioinformatics. 2017 Mar 22;33(14):2209–11. doi: 10.1093/bioinformatics/btx148 (PMC5870551; doi:10.1093/bioinformatics/btx148)

| **Supplementary Table 1.** Summary of available file format parsers in *ConKit* | | | |
| --- | --- | --- | --- |
| Contact prediction file formats | | Sequence file formats | |
| Format | Keyword | Format | Keyword |
| Bbcontacts | bbcontacts | A3M | a3m, a3m-inserts^a^ |
| BCLcontact | bclcontact |  |  |
| Casp RR | casprr | FASTA | fasta |
| CCMpred | ccmpred | Jones^b^ | jones |
| COMSAT | comsat | Stockholm | stockholm |
| EPCMap | epcmap |  |  |
| EVfold | evfold |  |  |
| FreeContact | freecontact |  |  |
| GREMLIN | gremlin |  |  |
| MemBrain | membrain |  |  |
| Pcons[C\|C2\|C3] | pconsc, pconsc2, pconsc3 |  |  |
| PDB | pdb |  |  |
| PlmDCA | plmdca |  |  |
| (Meta-) PSICOV | psicov, metapsicov |  |  |
| ^a^ The a3m-inserts keyword stores the alignment including insert states.  ^b^ This file format corresponds to one sequence per line without header information. | | | |

**Supplementary Figure 1.** An example 2-dimensional contact map visualised using *ConKit*’s plotting functionality. Example data based on PDB entry: 1DTX.


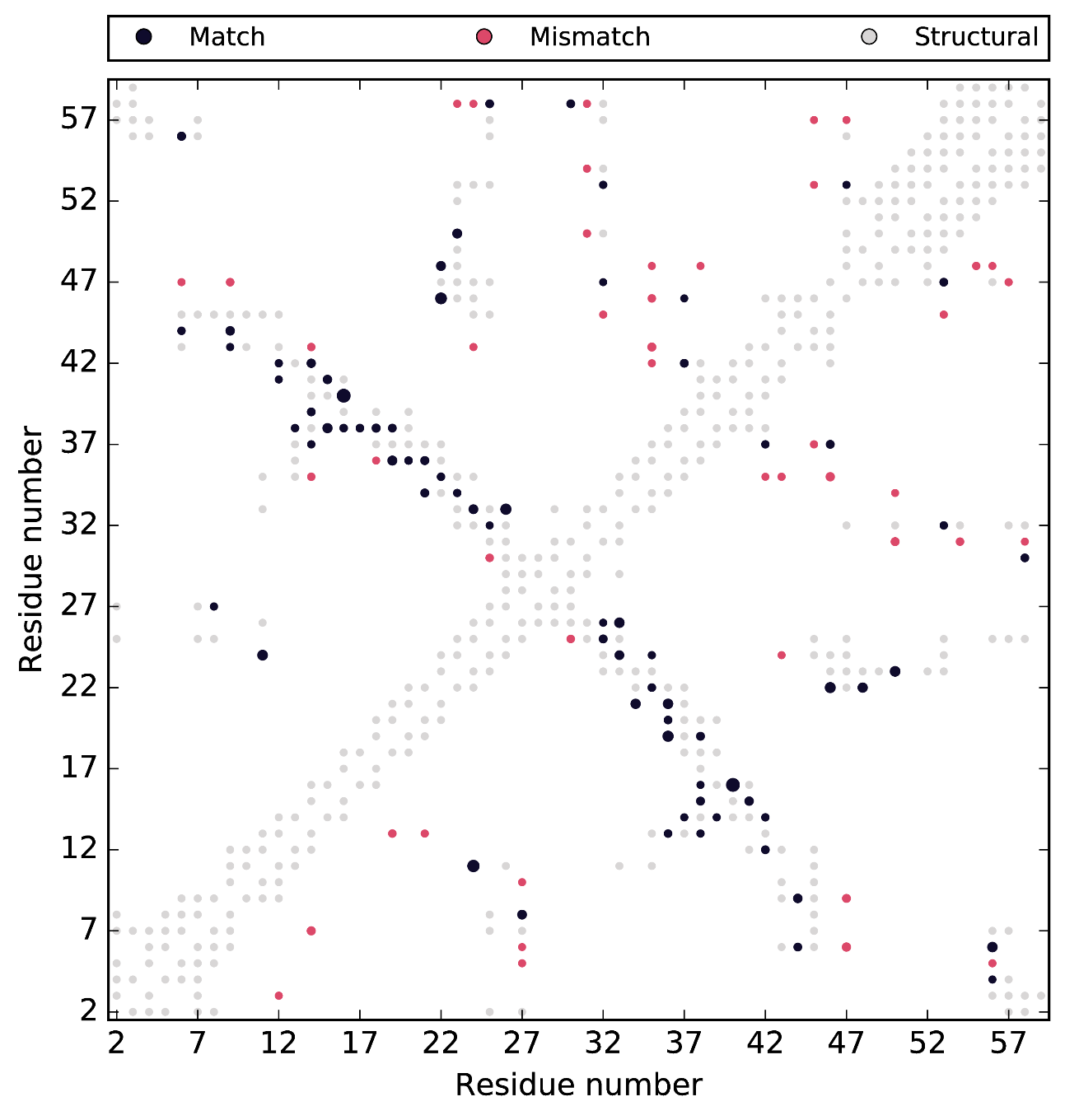

Supplement: Supplementary Data [file btx148_supp.docx]
